# Data-Enriched Edible Pharmaceuticals (DEEP) with Bespoke Design, Dose and Drug Release

**DOI:** 10.3390/pharmaceutics13111866

**Published:** 2021-11-04

**Authors:** Meie Chao, Heidi Öblom, Claus Cornett, Johan Bøtker, Jukka Rantanen, Sofia Kälvemark Sporrong, Natalja Genina

**Affiliations:** 1 Department of Pharmacy, University of Copenhagen, Universitetsparken 2, 2100 Copenhagen, Denmark; meiechao95@gmail.com (M.C.); heidi.oblom@abo.fi (H.Ö.); claus.cornett@sund.ku.dk (C.C.); johan.boetker@sund.ku.dk (J.B.); jukka.rantanen@sund.ku.dk (J.R.); sofia.kalvemark-sporrong@farmaci.uu.se (S.K.S.); 2 Pharmaceutical Sciences Laboratory, Faculty of Science and Engineering, Åbo Akademi University, Artillerigatan 6A, 20520 Åbo, Finland; 3 Department of Pharmacy, Uppsala University, P.O. Box 580, 751 23 Uppsala, Sweden

**Keywords:** patient-centered pharmaceutical design, additive manufacturing, inkjet printing, cannabinoids, polymer coating, QR code

## Abstract

Data-enriched edible pharmaceuticals (DEEP) is an approach to obtain personalized medicine, in terms of flexible and precise drug doses, while at the same time containing data, embedded in quick response (QR) codes at a single dosage unit level. The aim of this study was to fabricate DEEP with a patient-tailored dose, modify drug release and design to meet patients’ preferences. It also aimed to investigate physical stability in terms of the readability of QR code patterns of DEEP during storage. Cannabinoids, namely, cannabidiol (CBD) and delta-9-tetrahydrocannabinol (THC), were used as the model active pharmaceutical ingredients (APIs). Three different substrates and two colorants for the ink were tested for their suitability to fabricate DEEP by desktop inkjet printing. Flexible doses and customizable designs of DEEP were obtained by manipulating the digital design of the QR code, particularly, by exploring different pattern types, embedded images and the physical size of the QR code pattern. Modification of the release of both APIs from DEEP was achieved by applying a hydroxypropyl cellulose (HPC) polymer coating. The appearance and readability of uncoated and polymer-coated DEEP did not change on storage in cold and dry conditions; however, the HPC polymer layer was insufficient in preserving the readability of the QR code pattern in the extreme storage condition (40 °C and 75% relative humidity). To sum up, the DEEP concept provides opportunities for the personalization of medicines, considering also patients’ preferences.

## 1. Introduction

Currently, the majority of drug products available on the market can be classified as “one-size-fits-all”. This implies that most drug products are commercialized in a limited number of fixed doses to fit the largest portion of the target population without considering the individual’s specific needs [1]. A drug response to most of the marketed drug products ranges from 50% to 75%. However, it can vary from 10% to 90%, depending on the drug product. For example, 80% of patients are drug-responsive to COX-II inhibitors, such as nonsteroidal anti-inflammatory drugs (NSAIDs), whereas only 25% of cancer patients respond to chemotherapy [2]. Several factors, such as physiology, psychology, pharmacology, lifestyle and genetics of the patient, may influence the drug response [2,3,4]. Therefore, some patients experience inadequate treatment due to improper doses: either no therapeutic effect if the dose is too low, or adverse drug reactions (ADR) if the dose is too high [5].

Two subpopulations that are particularly susceptible to the lack of appropriate doses, dosage forms, and drug release kinetics are pediatric and geriatric groups [6]. This is because children and elderly are different, in regards to age and status of health, from the average and healthy “standard” person, whose response to the drug defines the recommended doses [7]. Both subgroups commonly also have difficulties in swallowing conventional dosage forms [8]. For example, more than 50% of children are physically unable to swallow a standard size pill or small capsule [9,10]. This can contribute to non-compliance and ineffective treatment [11], suggesting that there is an unmet need for more suitable dosage forms. Furthermore, elderly are commonly polypharmacy patients, i.e., they take at least five medicines [12]. Correct identification and following the prescribed administration regimen for each drug product by these polypharmacy patients can be challenging. 

Additive manufacturing (AM), also called pharmacoprinting when applied in the pharmaceutical field, has emerged as a promising new technology to provide personalized medicine on demand, i.e., the production of patient-tailored dosage form, dose, drug release kinetics, when there is a clinical need [1,13,14]. AM is an umbrella term that includes various 2D and 3D printing techniques, where the design and manufacturing process of dosage forms are digitally well controlled, in contrast to many other drug manufacturing technologies [15]. Pharmacoprinting has been explored for customization of dosage forms to meet the patient’s preferences and needs [16,17,18,19]. Furthermore, printing techniques, e.g., inkjet printing, allow fabrication of dosage forms with unique designs, e.g., quick response (QR) code pattern, readable by a standard smartphone, thereby providing extra functionality and allowing further individualization of the dosage form [20,21,22]. 

Data-enriched edible pharmaceuticals (DEEP) have been proposed as personalized dosage forms for oral drug delivery, where the patient-tailored dose is printed in the pattern of a unique QR code that encapsulates relevant information [23]. The QR code pattern can be used to ease identification of the dosage form, obtain patient-tailored or dosage form–tailored information (e.g., patient information sheet with embedded videos, regarding the correct administration of drug products) and allow for traceability of the drug product at a single dosage unit level, even if the original package is lost or not available [6]. DEEP was fabricated using an orodispersible formulation, thereby minimizing the swallowing difficulties. Flexible doses in DEEP have been achieved by changing the physical dimensions of the QR code patterns [20] or the printing of multiple subsequent layers [23]. Modification of the drug release properties of DEEP, digital dose adjustments and the customization of DEEP’s appearance have not yet been investigated. The aim of this study was to fabricate DEEP that would meet the patients’ preferences in regard to color, size and design as well as provide easily adjustable doses and release profiles, while still preserving the readability of the QR code patterns during storage. 

## 2. Materials and Methods

### 2.1. Materials

The drug-containing ink for inkjet printing was formulated based on the oromucosal drug product Sativex^®^, containing 27 mg/mL delta-9-tetrahydrocannabinol (THC) and 25 mg/mL cannabidiol (CBD) that was obtained from 2care4 (Esbjerg, Denmark). The coloring agents, erythrosine B (90% dye content) and brilliant black BN (60% dye content), were purchased from Sigma-Aldrich (St. Louis, MO, USA). The placebo ink was based on ethanol (96%) from VWR (Fontenay-sous-Bois Cedex, France) and propylene glycol (PG) from Sigma-Aldrich (≥99.5%, Sigma-Aldrich, St. Louis, MO, USA). For the preparation of the orodispersible substrates (solid foam), hydroxypropyl methylcellulose (HPMC) (Metolose 60SH-4000) (Shin-Etsu, Tokyo, Japan), poloxamer 188 (Lutrol^®^ F68) (Sigma-Aldrich, Steinheim, Germany), polyethylene glycol 4000 (PEG 4000), Merck KGaA, Darmstadt, Germany), polysorbate 20 (Tween^®^ 20) (Merck KGaA, Fontenay-sous-Bois Cedex, France) and glycerol (≥99%, Sigma-Aldrich, Petaling Jaya, Malaysia) were used. The transparent laser paper (type A paperbacked laser/copier transparencies) used for casting polymer dispersion was from Xerox (Norwalk, CT, USA). Potato starch substrate (wafer paper) (AB Marketing GmbH, Ebern, Germany), fondant paper (A4, AB Marketing GmbH, Ebern, Germany) and normal copy paper (A4. 80 g/m^2^, Plano^®^ Universal, EU) were used as the commercially available substrates for printing. Hydroxypropyl cellulose (HPC) (150–400 mPa s for 2% aqueous solution) (Tokyo Chemical Industry Co., Ltd, Toshima, Kita-Ku, Tokio, Japan) was used as the polymer for coating. For the analyses with ultra-high-performance liquid chromatography (UHPLC), acetonitrile was purchased from VWR Chemicals (Fontenay-sous-Bois Cedex, France), and Milli-Q water was freshly prepared with Ultrapure (Type 1) Water (Merck KGaA, Darmstadt, Germany). For the artificial saliva, sodium chloride, potassium dihydrogen phosphate, and disodium hydrogen phosphate (Merck KGaA, Darmstadt, Germany) were used.

### 2.2. Ink and Substrate Preparation

The ink for printing was made by spraying 5 mL Sativex^®^ in a glass vial and adding either erythrosine B or brilliant black BN in the concentration of 10 mg/mL. The ink was injected into the empty refillable cartridge suitable for an Epson XP-8500 desktop piezoelectric inkjet printer (Seiko Epson Corporation, Nagano, Japan) with a syringe without a filter. 

The orodispersible substrate was prepared in the following way: a 100 mL polymer solution was made by slowly dispersing 2.50 g of HPMC, 0.0825 g of Lutrol^®^ F68, 0.25 g of PEG 4000, 0.25 g of Tween 20 and 0.25 g of glycerol into 50 mL of preheated (70 °C) Milli-Q water. The mixture was stirred on a magnetic stirrer for 5 min before adding further 50 mL Milli-Q water (~20 °C) with continuous magnetic stirring. The aqueous dispersion was stirred for about 30 min until a clear viscous dispersion was obtained. In order to remove the entrapped air bubbles, the polymer dispersion was kept in a refrigerator at 2–8 °C for at least 24 h. The formulation was then cast on a transparent laser paper with a 3D-printed casting knife with a gap size of two millimeters. The paper sheets were cut to fit the shelves of an Epsilon 2–4 LSC freeze dryer (Martin Christ, Osterode, Germany). The freeze-drying program was set according to the method described in a previous study by Öblom et al. [23]. The thickness of the used substrates was measured with a digital caliper at five different locations.

### 2.3. Inkjet Printing of DEEP

QR codes (linking to a URL) with different patterns were generated by the website, www.qrcode-monkey.com, and downloaded as .png files. The images were resized to 2.0 cm^2^ and 2.2 cm^2^ in PowerPoint (Microsoft Office, Albuquerque, NM, USA). The prepared substrates were cut in 2.5 cm by 10.0 cm strips, taped on a sheet of copy paper (A4 format), and loaded into the rear paper feed slot of the printer. Four QR codes were printed at a time, and they were set to dry for up to 2 min before printing the next layers. The printed DEEP were kept in plastic bags with zip locks and stored in a desiccator with silica in the fridge at 5 °C for further analysis. The final DEEP were cut from a size range of 2 × 2 cm^2^ and 2.2 × 2.2 cm^2^. The commercial substrates were used without any modifications, using the same printing procedure as described above. 

### 2.4. Readability of DEEP

Normal copy paper, starch paper, fondant paper and the prepared freeze-dried substrate were used to explore the readability of the printed QR codes. Various numbers of layers were printed on top of each other to showcase the feasibility of manipulating the dose. After each printed layer, the readability was checked with a smartphone camera (iOS 13.7) and a code scanner app, scan.me (version 2.8).

Pixel calculations of the QR patterns were done in MATLAB R2017b (MathWorks, Natick, MA, USA), using the imbinarize function and subsequent summation (Appendix A).

### 2.5. Coating of DEEP

A 1% (*w*/*v*) solution of HPC in ethanol was used for coating the DEEP with an airbrush (Model BD-134, Fengda), having a nozzle diameter of 0.2/0.3 mm at a pressure of 2 bars and at a 20 cm distance from the DEEP for 20 s.

### 2.6. Drug Content Analysis

For a standard curve, a stock solution was made by mixing 40 µL Sativex^®^ and 960 µL of the diluent, consisting of a mixture of acetonitrile and water (70:30) with 0.1% formic acid (FA) added. The concentration of CBD and THC in the solution were 1.00 mg/mL and 1.08 mg/mL, respectively. The standard curve was prepared in concentrations ranging from 0.0067 mg/mL to 0.4000 mg/mL for CBD, and 0.0072 mg/mL to 0.4320 mg/mL for THC. The limit of detection (LOD) was 0.0092 mg/mL and 0.0076 mg/mL for CBD and THC, respectively. The limit of quantification (LOQ) was 0.0230 mg/mL and 0.0279 mg/mL for CBD and THC, respectively.

DEEP with various numbers of subsequently printed layers and different patterns were put in glass vials and dissolved in 3 mL of the diluent (*n* = 3). The glass vials were vortexed until the DEEP were dissolved and subsequently centrifuged with Hettich EBA20 (Tuttlingen, Germany) at 5000 rpm for 10 min. Afterward, 200 µL of the supernatant was transferred to silanized vials with 450 µL silanized inserts (Phenomenex, Værløse, Denmark) and enclosed with 11 mm combination seals (Mikrolab Aarhus A/S, Højbjerg, Denmark). For the DEEP imprinted with seven layers, 100 µL of the supernatant was diluted with 100 µL of diluent to achieve a concentration suitable for the range of the standard curve. 

The standard curves and content analysis were performed on an Ultimate 3000 ultrahigh-performance liquid chromatography (UHPLC) from Thermo Scientific (Waltham, MA, USA) with a Kinetex, C18 column of 1.7 µm, 100 × 2.1 mm (Phenomenex, Værløse, Denmark), detection at 228 nm, using a slightly modified method from Öblom et al. [23]. The vacuum degasser and column compartment were set to have a nominal temperature of 30 °C, and the autosampler was set to have a nominal temperature of 8 °C. Two mobile phases, A and B, were used. Mobile phase A was prepared by adding 0.1% FA to Milli-Q water, and mobile phase B was prepared by adding 0.1% FA to acetonitrile. Both mobile phases were placed in a Branson 2210 ultrasonic cleaning bath from Marshall Scientific (CT, USA) for 10 min. A multistep gradient program on the UHPLC was set to initially consist of 30% mobile phase A and 70% mobile phase B for the first 6 min, followed by an increase of 100% mobile phase B, which was kept constant from 6 to 8 min. Between 8 and 8.1 min, mobile phase B was decreased to 70%, followed by equilibration of the column with the initial conditions until 11 min. The flow rate and sample injection volume were 0.3 mL/min and 10 µL, respectively. The software Thermo Xcalibur and Excel 2020 (Microsoft) were used for the data analysis. 

### 2.7. Drug Release Studies

Artificial saliva was made by adding 4.00 g sodium chloride, 0.95 g potassium dihydrogen phosphate, 1.19 g disodium hydrogen phosphate, and 2.50 mL Tween^®^ 20 to 500 mL Milli-Q water. The solution was adjusted to pH 6.8 with 1 M hydrochloric acid. 

The artificial saliva was heated to 37 °C in a water bath containing distilled water. Coated and uncoated DEEP imprinted with seven layers were put in glass vials, filled with 6 mL of the heated artificial saliva and placed in the water bath (*n* = 3). After 5, 10, 30, 60, 120, and 180 min, 200 µL samples were withdrawn and transferred to 1.5 mL Eppendorf tubes, and 200 µL of artificial saliva was refilled into the glass vials. The Eppendorf tubes were centrifuged at 4 °C at 10,000 rpm for 10 min. For analysis, 150 µL of the supernatant samples were transferred to silanized vials with silanized inserts, enclosed with seals and analyzed on the UHPLC. 

### 2.8. Long-Term Stability

Coated and uncoated DEEP were put in desiccators at 40 °C (75% RH) and 5 °C (silica) (*n* = 3). The readability of DEEP was checked every week for 6 weeks and after 10 months. 

### 2.9. Statistical Analysis

The statistical analysis was conducted using *t*-Test: Two-Sample Assuming Equal Variance in Microsoft Excel. A significance level of 5% was used.

## 3. Results and Discussion

### 3.1. Fabrication of DEEP

**Figure 1:**
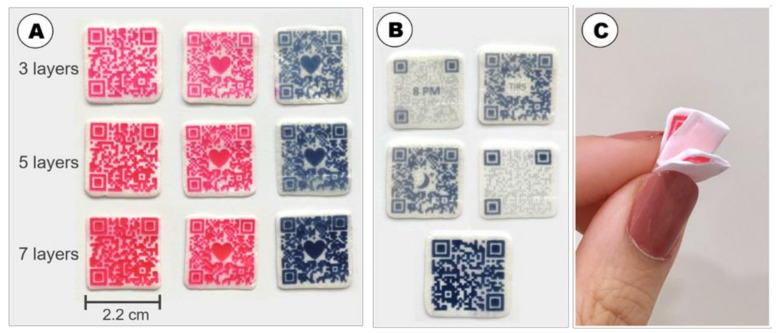
Photographs of (**A**) inkjet-printed DEEP, using solid foams as substrate with three, five and seven subsequently imprinted layers, where the ink contained either red or blue coloring agents; (**B**) inkjet-printed DEEP with different patterns (original and dots), incorporated images (moon and text; TIRS = Tuesday), and sizes (2 × 2 cm^2^ and 2.2 × 2.2 cm^2^) (top four DEEP are imprinted with 3 layers and bottom DEEP is imprinted with 7 layers). (**C**) Flexibility of an inkjet-printed DEEP.

The preparation of DEEP by inkjet printing requires an ink in the printable range in regards to optimal viscosity, surface tension and solubility of the solid ingredients (i.e., APIs, colorants) in the ink [24]. In addition, the fabrication of DEEP demands a substrate that is compatible with the ink and that possesses properties which enable efficient absorption of the ink without it being disintegrated and dissolved. Furthermore, the printed pattern on the substrate should have minimal ink bleeding and/or dye migration to ensure good print edge definition. This is of great importance when dosage forms, such as DEEP, are printed in the pattern of a QR code to ensure its readability by a standard smartphone. In this study, the ink, containing cannabinoids, was formulated in a similar way as reported by Öblom et al. [23]. However, two coloring agents were tested in the ink formulation: either (a) red colorant (erythrosine B) or blue colorant (brilliant black BN) (Figure 1A). These particular dyes were chosen (a) because patients preferred DEEP, either in blue or red color, according to a recent study [25], and (b) to make a solution-type ink (both colorants dissolved in the ink) to avoid clogging of the firing nozzles in the printer. Both inks contained colorants in a high concentration to make sure that the printed QR pattern would be visible and readable by a standard smartphone. The amount of the coloring agent (erythrosine B) in a DEEP was within the acceptable daily intake [26,27,28].

A good ink absorption capacity of the substrate is important to enable incorporation of a sufficient volume of the ink in order to reach the therapeutic doses of the APIs in DEEP [29]. It also prevents accumulation of the printed ink at the surface of the substrate that otherwise would result in smearing the ink during handling or further printing if a contact printer is used. Therefore, three different substrates were used in this study, namely, commercially available fondant paper, starch paper and in-house prepared highly porous substrates (solid foam), to evaluate their ability to absorb and entrap the ink by printing multiple layers on top of each other. The thickness of the substrates were 1.01 ± 0.06 mm, 0.34 ± 0.02 mm, and 1.55 ± 0.03 mm for fondant paper, starch paper and solid foam, respectively. The studied fondant paper and starch paper could not be imprinted with more than three layers, due to their low absorption capacity and the observed smearing that made recognition of a QR code by a smartphone impossible. The low absorption of these two substrates is obviously a consequence of their low porosity [23,24]. Bleeding of ink from the back side was not observed for these two substrates, as the imprinted ink remained mainly on the surface of the substrates. On the contrary, the solid foam of high porosity [23,30] could be imprinted with up to 14 subsequent layers, and the QR code pattern was still readable. However, from the 8th layer, the substrate was soaked, resulting in visible ink on the back side of the substrate. A thicker substrate was prepared to investigate whether it would be possible to print additional layers without the ink leaking from the back side of the substrate. However, the commercial contact printer used in this study had a tendency to compress the substrate by the rollers [23] when the critical thickness of the substrate was reached. Therefore, the ink bleeding on the back side and ink smearing were visible, even with the thicker substrate, underlining the need for the use of non-contact inkjet printers if a higher number of imprinted layers is desired. In this study, the commercial desktop inkjet printer was used, because it was inexpensive and allowed fast printing times. The printing time was 14.0 ± 0.1 s for one DEEP and 19.2 ± 0.1 s for four DEEP located horizontally in the same row. Hence, the development of printing equipment for pharmaceutical use is needed to allow easier implementation of a new technology for manufacturing drug products that can meet the existing strict quality control requirements [6]. 

In addition, the mechanical properties of substrates, and eventually DEEP, are important to avoid their damaging during printing, packaging and handling. It appeared that DEEP based on solid foams were easily bendable without any visible damage (Figure 1C), whereas both fondant paper and starch paper would crack during folding.

A QR code pattern can have different physical appearance and can also be enriched with different images for better recognition and customization. It was possible to print DEEP with different QR pattern types, original and dots (rasterized), to incorporate different images (heart, moon and text) and to prepare different physical sizes (2 × 2 cm^2^ and 2.2 × 2.2 cm^2^) (Figure 1A,B). The chosen patterns, incorporated images and physical size of the DEEP were based on the results of a recent study, where patients’ preferences for the design of DEEP were investigated [25].

### 3.2. Drug Content

**Figure 2:**
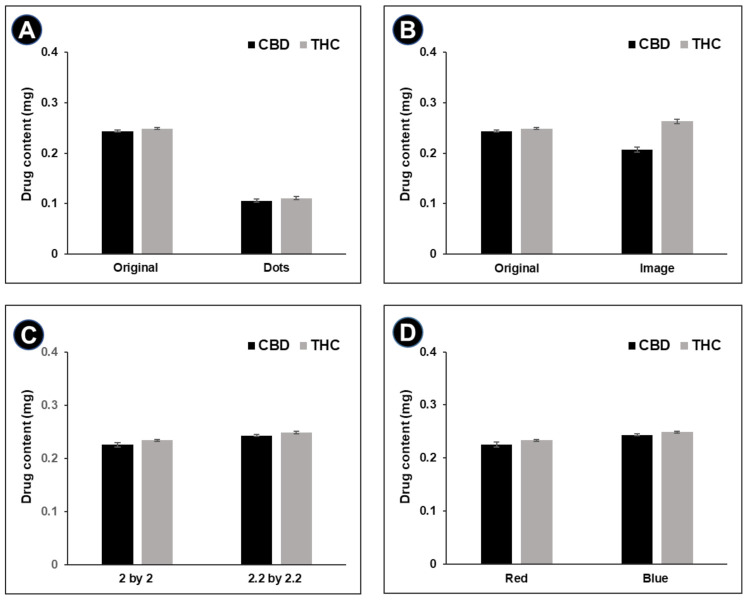
CBD and THC content of DEEP of (**A**) different patterns (original and dots (rasterized), printed in blue), (**B**) with and without the embedded image (original and ‘heart’ image, printed in blue), (**C**) different physical sizes (2 × 2 cm^2^ and 2.2 × 2.2 cm^2^, printed in red) and (**D**) containing different coloring agents (printed in red and blue). Data are presented as average ± SD (*n* = 3). All compared samples were significantly different (*p* < 0.05).

The pattern type, incorporated image, and size of the QR code (Figure 1A,B) affect the amount of the ink printed in DEEP. Digital manipulation of these parameters allows personalization of the dose in an easy way, compared to mechanical and manual adjustment required for batch production with conventional manufacturing techniques. Therefore, each parameter at a time was varied in the digital design of a QR code pattern in order to identify its influence on the drug content of the DEEP. It was expected that a higher print density (a higher pixel count of the QR code pattern) would give a higher printed volume of the ink at same print area, which would subsequently result in a higher dose [31]. This was proven by comparing the dose of both CBD and THC for original and dotted QR code patterns, where the original QR code pattern contained 2.1 times more pixels, compared to the dotted pattern (Table 1). The UHPLC analysis showed, indeed, that DEEP with a dotted QR code pattern contained less than half the amount of both APIs as compared to DEEP with an original QR pattern (the amount of printed layers and the physical size were the same for both QR code patterns) (Figure 2A). Incorporation of an image, such as a heart, in DEEP resulted in a slight increase in the pixel count that was reflected in the increased content of THC in the same range as compared to the DEEP with an original pattern (Figure 2B). The CBD content of DEEP with an embedded pattern was inconclusive, most obviously due to an analytical error. There was a significant difference in the drug content when the physical dimensions of DEEP were varied (*p* = 0.0033 and 0.0001 for CBD and THC, respectively). For the DEEP imprinted with five layers, an increase of 0.2 cm/side of the QR code pattern resulted in a mean increase of 7.2% and 5.9% for CBD and THC, respectively (Figure 2C). This was in accordance with the colored pixel count that was present in the bigger QR code pattern, compared to the original pattern (Table 1). The dose adjustment in a dosage form by digital manipulation of the printed area was previously observed by other researchers [20,32,33]. In the future, both the designs of QR codes and the correction of the number of pixels should be done with an algorithm that counts the pixels and adjusts their number by resizing the QR code pattern and/or adding special types of symbols to ensure that the desired dose of APIs is printed in a DEEP. It is worth noting that the DEEP presented in this study are not meant to be manually divided to reduce, for example, a dose, because the drug distribution is different within a single DEEP. In other words, the cutting of DEEP into equal parts would not guarantee the same amount of APIs in each part. However, the content of both APIs in each DEEP is the same. Moreover, it was seen that the DEEP printed with brilliant black BN dye (blue) had a significantly higher amount of both APIs than DEEP printed with erythrosine B (red) (*p* = 0.0028 and 0.0001 for CBD and THC, respectively) (Figure 2D). This indicates that the presence of colorants could affect the drug content.

**Figure 3:**
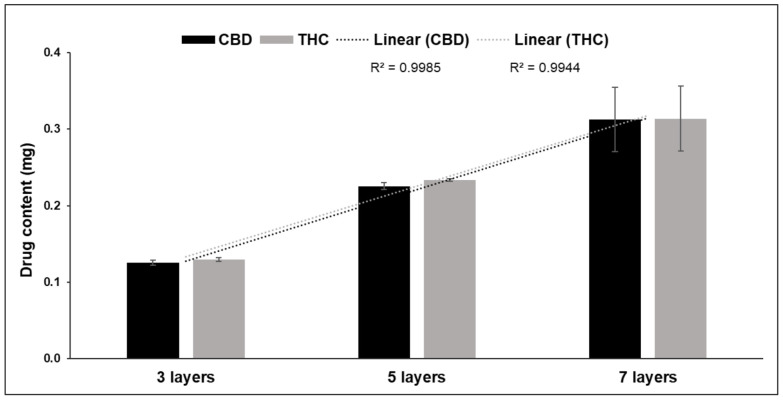
CBD and THC contents of DEEP with three, five and seven imprinted layers. Data are presented as average ± SD (*n* = 3).

There was a linear correlation between the number of printed layers and the cannabinoid content (R^2^ = 0.998 for CBD, and R^2^ = 0.994 for THC) (Figure 3). The average amount of CBD per printed layer was 0.041 ± 0.01 mg, and 0.043 ± 0.01 mg for THC. The average ratio for THC to CBD was 1.06. This means that, on average, 1.6 µL of the ink was printed per layer. The positive correlation between the drug content and the number of layers printed was shown by Öblom et al. [23]. However, in this study, the achieved drug content for the three, five and seven layers was slightly lower than reported by Öblom et al., despite using the same method. This might be explained by the differences in the printing conditions, as the amount of the printed ink could vary depending on the cartridges and condition of the firing nozzles. It could also be that the colorants caused partial clogging of the nozzles, resulting in less ink being ejected [20], and potentially a larger standard deviation in the dose. Furthermore, some smearing of the ink with a high number of the printed layers (high standard deviation for DEEP with seven imprinted layers) could happen as a result of incomplete absorption of the ink onto the substrate, due to insufficient time between printing subsequent layers and the use of a contact printer. None of the fabricated DEEP had a therapeutic dose of either CBD or THC. This is because the ink used was based on the market available product with low concentrations of CBD and THC, due to difficulties in obtaining raw APIs. As the printed volume of ink per layer is very small, the formulation of ink with a higher concentration of the APIs, close to the solubility limit in the ink base, would help to increase the dose. This approach was used before by having highly concentrated ink formulations [30,34].

The ratio between the two cannabinoids in the DEEP (1.06) was very similar to one in the original drug product, Sativex^®^ (1.08). This indicates that the ratio of cannabinoids was not affected by the process of inkjet printing. The outcomes of the drug content analysis suggest that inkjet printing of DEEP using desktop printers can be used to prepare flexible doses of APIs by simple manipulation of the digital design of the pattern to be printed, though it is more suitable for low dose APIs, as also pointed out by other studies [22,35].

### 3.3. Drug Release

**Figure 4:**
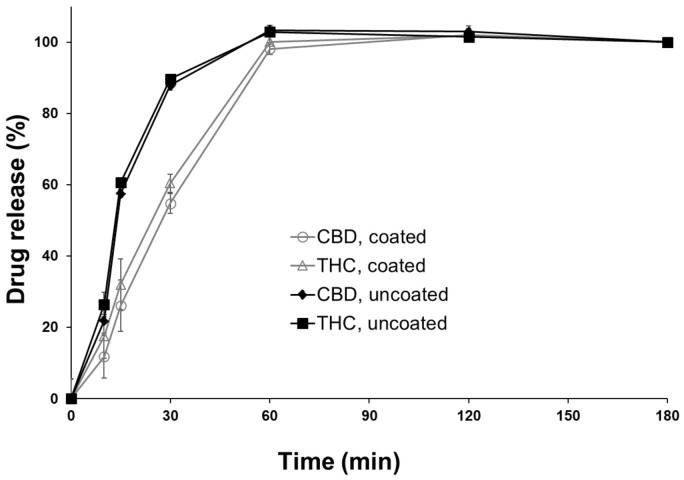
Drug release of CBD and THC of coated and uncoated DEEP with 7 imprinted layers. Data are presented as mean ± SD (*n* = 3).

The purpose of applying a polymer coating can be to modify the release rate of an API from the dosage form [36]. Hydroxypropylcellulose (HPC) polymers of various grades have been used to tailor the release rate of APIs [37]. In this study, a thin HPC coating (low viscosity grade) was used to investigate whether it could modify the release of both APIs from DEEP. It was observed that the drug release rate from the coated DEEP was slower than the drug release from the uncoated DEEP (Figure 4). The amount of drug released from the uncoated DEEP was higher during the first 60 min of in vitro testing when compared to the amount released after 120 min, where the full drug amount was released from both the coated and uncoated DEEP. Obviously, a thicker coating layer and the choice of a higher viscosity grade HPC or another polymer could even further prolong the release rate of APIs from DEEP. Genina et al. successfully used ethylcellulose as a polymer for coating at different thickness levels to modify the release rate of the inkjet-printed APIs [38]. The ethylcellulose coating provides an extended release for APIs, though it is insoluble in water; therefore, it could be unsuitable for orodispersible dosage forms. 

### 3.4. Long-Term Physical Stability

Polymer coating of solid dosage forms is also performed to avoid the direct contact with an API during handling, for taste masking and/or protection against environmental conditions, such as temperature and moisture [39]. The DEEP were coated with a HPC protective layer to investigate the physical stability of DEEP, particularly the readability of QR code patterns upon storage, as previous research revealed readability issues when uncoated DEEP were stored in a warm and humid environment [23]. The main reason for choosing the HPC polymer was that it forms a transparent film upon drying from both aqueous and alcoholic solutions (dispersions), which is important to ensure the readability of QR codes in DEEP after coating. In addition, it is soluble in water, which is important when orodispersible formulations are designed.

**Figure 5:**
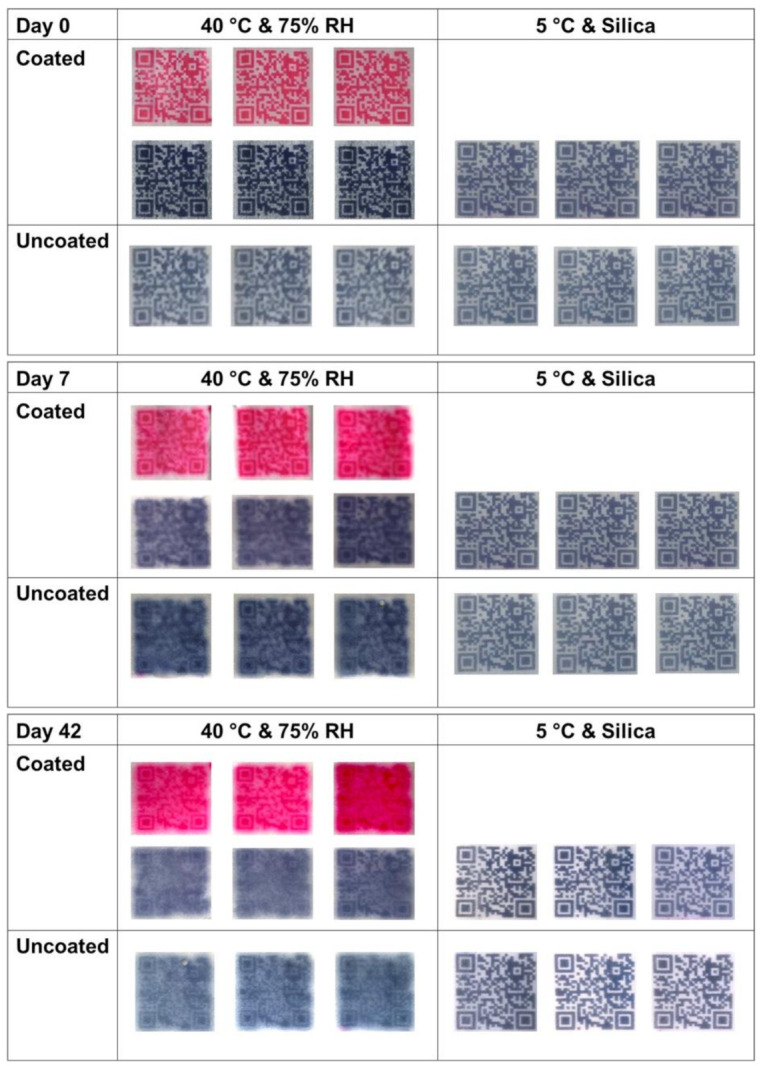
Photographs of DEEP, uncoated and coated, stored in two different conditions at 0 days, 7 days and 42 days of storage.

In this study, all the DEEP (coated and uncoated) stored in a cold and dry environment (5 °C, silica) were still readable by a standard smartphone after 10 months of storage. None of the DEEP stored in the warm and humid environment (40 °C, 75% RH) were readable after seven days of storage, due to dye migration and, consequently, a poor edge definition of the QR code patterns (Figure 5). Even though none of them were readable, the DEEP printed with erythrosine B seemed to have a better print edge definition and better contrast between the QR code pattern and the background than the DEEP printed with brilliant black BN, suggesting that erythrosine B is a preferable colorant to be used for DEEP.

The stability studies revealed that the HPC coating could not provide sufficient protection for DEEP with cannabinoids against harsh storage conditions, suggesting that either a thicker coating, another way of coating or another type of coating should be used. For example, non-hygroscopic/low water activity excipients, particularly the film-forming polymers with the lowest amount of inherent moisture, would be a rational choice [39]. 

Furthermore, it seems that the storage stability of a DEEP is API dependent, as the previous study revealed a more stable DEEP with haloperidol as the API, even though it was stored in conditions of higher relative humidity [20]. This stability study confirms the importance of storage and packaging of DEEP, suggesting that DEEP containing cannabinoids should be stored in cold and dry conditions, e.g., in sealed package and in a refrigerator to ensure a longer shelf life. 

Despite the fact that the applied coating could not sustain readability of the DEEP when they were stored at 40 °C and 75% RH, the physical appearance and readability of the DEEP did not change after storage in cold and dry conditions for 10 months (data not shown). This indicates that the polymer coating can still act as a barrier to avoid direct contact with the API during handling. Furthermore, it could still potentially provide the initial taste masking functionality and, importantly, avoid stains of the dye on the tongue after administration of DEEP. Staining of the tongue was one of the concerns pointed out by patients when the DEEP concept was discussed [25].

## 4. Conclusions

It was possible to fabricate DEEP containing cannabinoids, using a desktop inkjet printer with two colors, namely red (erythrosine B) and blue (brilliant black BN), onto three different substrates. The red colorant could be considered superior, as it gave better print edge definition and better contrast between the QR code pattern and the background (substrate). Solid foam appeared to have the best ink absorption capability from the studied substrates. DEEP containing flexible doses were achieved by digitally varying the pattern type, embedded image and the physical size of the QR code. The dose was increased by increasing the print density and the size of the DEEP. It was also possible to linearly increase the dose of the APIs in DEEP by increasing the number of imprinted layers. The coating of DEEP with HPC polymer film resulted in a prolonged drug release of CBD and THC but did not ensure the readability of the DEEP in a warm and humid environment during storage. On the contrary, DEEP stored in a cold and dry environment were still readable after 10 months. This study suggests that the coating of DEEP could still act as a barrier, avoiding direct contact with the API during handling.

## Figures and Tables

**Table 1:** The pixel counts of digital designs of the printed patterns.

Image of Digital Design	Digital Design	Total Pixel Count	Colored Pixel Count	Pixel Ratio to Original QR Code Pattern	Ratio in Content of CBD/THC to Original QR Code Pattern, *n* = 3
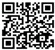	Original QR code pattern, 2 × 2 cm^2^	12,769	6480	1.00	1.00/1.00
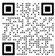	Dotted QR code pattern, 2 × 2 cm^2^	12,656	3053	0.47	0.43/0.44
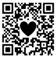	QR code pattern with embedded ‘heart’ image	12,656	6697	1.03	0.85/1.06
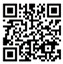	QR code pattern, 2.2 × 2.2 cm^2^	15,376	7656	1.18	1.08/1.06

## Data Availability

Not applicable.

## Appendix A

MATLAB code for pixel calculations:

clear

close all

disp(‘Choose photo to be analyzed’)

master=uigetfile(‘*’);

fileName=master(1:end-4);

info=imfinfo(master);

if size(info.Colormap)>1

[im, map]=imread(master);

BB=ind2rgb(im,map);

BB=rgb2gray(BB);

BB=imcomplement(BB);

BBB=imbinarize(BB);

BBB=double(BBB);

figure;imagesc(BBB)

else

im=imread(master);

BB=rgb2gray(im);

BBB=imbinarize(BB);

BBB=imcomplement(BBB);

figure;imagesc(BBB)

title(fileName)

end

[m, n, o] =size(BBB);

QR_code_pixels = sum(sum(BBB))

Total_number_of_pixels_in_image = m*n

Pixel_size_of_image= [m n o]
